# Overweight, stunting, and concurrent overweight and stunting observed over 3 years in Vietnamese children

**DOI:** 10.1080/16549716.2018.1517932

**Published:** 2018-09-26

**Authors:** Loan Minh Do, Lauren Lissner, Henry Ascher

**Affiliations:** a Outpatient Department, National Children’s Hospital, Hanoi, Vietnam; b Section for Epidemiology and Social Medicine (EPSO), Department of Public Health and Community Medicine, Institute of Medicine at the Sahlgrenska Academy, University of Gothenburg, Gothenburg, Sweden; c Research Department, Angered Hospital, Gothenburg, Sweden

**Keywords:** Overweight, stunting, concurrent overweight and stunting, preschool children, longitudinal study, Vietnam

## Abstract

**Background**: Malnutrition, both stunting and overweight/obesity, present a public health concern in many countries in the world.

**Objective**: This study aims to examine: (1) longitudinal changes in prevalence of overweight, stunting, and concurrent overweight and stunting among preschool children during 3 years and (2) secular changes in these prevalences of a specific age group of children aged 5.5–6.5 year over a period of 3 years.

**Methods**: A cohort of 2,602 children initially aged 3–6 years old, 1,311 in an urban area and 1,291 in a rural area, was followed for 3 years. Of them, children aged 5.5–6.5 years old were identified to be included in three repeated cross-sectional surveys. The World Health Organization standard was used to classify children with overweight or stunting.

**Results**: Findings from the cohort study indicate that between 2013 and 2016, the estimated prevalence of overweight including obesity (OWOB) increased with age, particularly in the urban setting (14.2%–29.9% in boys and 9.0%–21.6% in girls). The estimated prevalence of stunting decreased from 8.2% to 3.4% in boys and 9.5% to 3.5% in girls with a considerably greater decrease among rural children. There was a similar pattern of an age-related decrease of concurrent OWOB and stunting from 2.4% in 2013 to 1.4% in 2016 in boys and from 2.9% to 1.3% in girls with significant decreases in rural children. Secular trends in the group of children 5.5 to 6.5 show the same pattern as the longitudinal results: decreasing prevalence of stunting as well as concurrent OWOB and stunting. OWOB prevalence increased significantly in urban girls and rural boys.

**Conclusions**: The pattern of increasing overweight, decreasing stunting and concurrent overweight and stunting both with increasing age and over chronological time is observed among Vietnamese preschool children.

## Background

Malnutrition, both undernutrition and overnutrition, constitute a public health concern in many countries. The World Health Organization estimated that 161 million children under the age of five exhibited signs of stunted growth in 2013. About half of them live in Asia and over one-third in Africa [1]. At the same time, more than 42 million children under 5 years of age are overweight with the overwhelming majority – about 31 million – living in low- and middle-income countries [2]. Globally, between 1990 and 2016, the prevalence of stunting decreased in children under five from 39.5% to 22.9% [3]. Meanwhile, a secular trend in overweight in the same age group was observed, with a prevalence increase from 4.9% to 6.0% [3].

It has increasingly been observed that children can be overweight and stunted simultaneously. An Indonesian study reported in 2007 that the prevalence of concurrent overweight and stunting among children aged 2 to 4.9 year was 7.2% [4]. A similar study in Mexican indigenous children of the same age in 2003 found a prevalence of 10% [5]. Findings from a study in Limpopo province of South Africa reported that 31% of stunted children at 3 years of age were overweight and 40% of the overweight children were stunted [6].

The recently observed pattern of decrease in stunting [7] and increase in overweight in Vietnamese children [8] is similar to the global trend. However, concurrent overweight and stunting has not been previously explored. The aims of this study were to examine: (1) longitudinal changes in prevalence of overweight, stunting, and concurrent overweight and stunting among preschool children during 3 years and (2) secular changes in these prevalences of a specific age group of children aged 5.5–6.5 years over a period of 3 years. This paper presents results from longitudinal and repeated cross-sectional studies conducted in one urban and one rural area of Hanoi, Vietnam.

## Methods

### Study settings

The settings for this study were two Health and Demographic Surveillance Sites (HDSS), DodaLab and FilaBavi. DodaLab (about 11,000 households and 38,000 people) is located in the urban Dongda district and FilaBavi (about 11,000 households and 51,000 persons) in the rural Bavi district of Hanoi, Vietnam. The establishment of a HDSS provides a research setting for various studies as they provide demographic, economic and other information at the community level. The reported income per capita in 2013 in DodaLab and FilaBavi were USD 1,750 and USD 1,000 respectively. Details of the study sites were published previously [9].

### Study design, participants and data collection

A longitudinal study was designed to follow a cohort children aged 3–6 years old in 2013 living in strategically selected communes for 3 years. The number of recruited children was 2,842, 1,482 in the urban site and 1,360 in the rural site. The first measurement in 2013 was performed for 2,677 children, 1,364 urban and 1,313 rural by eight field workers in DodaLab and 12 in FilaBavi working in pairs at children’ homes. Digital Tanita scale and mobile stadiometers were used to measure each child’s weight and height. The field workers were specifically trained to work with young children, with special focus on how to measure weight and height. Anthropometric measurements of this cohort were identically repeated in 2014 and 2016. At the end of the study in 2016, 2,602 children (1,311 urban and 1,291 rural children) underwent three measurements and were used in the data analysis.

Among the 2,602 followed participants, all children aged 5.5 to 6.5 years in 2013, 2014 and 2016 were considered as three independent samples forming a repeated cross-sectional study. The numbers of children in the three samples were 546, 897 and 512 respectively.

Children were classified as overweight or stunted in accordance with the definitions of the WHO reference [10]. Concurrent overweight and stunting was defined when children were both overweight and stunted.

### Statistical analysis

Conventional statistical methods were used to estimate prevalence with confidence limits. To compare prevalences between ages within the cohort and its sub-cohorts, X^2^ tests for comparing proportions in dependent samples were used. For the comparisons of prevalences at different years for the 5.5 to 6.5 old children standard X^2^-test for comparison of independent samples were used.

## Results

Children participating in the longitudinal study and the cross-sectional surveys are described in Table 1. The percentages of boys and girls in both urban and rural sites were similar in the longitudinal study and the cross-sectional surveys.

**Table 1.** Distribution of the children participating in the longitudinal study and the cross-sectional surveys.

	Total	Mean age ± SD	Urban boys (n; %)	Urban girls (n; %)	Rural boys (n; %)	Rural girls (n; %)
Cohort of children 3 to 6 years old in 2013 participating in the longitudinal study						
Starting year 2013	2,602	4.6 ± 0.9	699 (26.9%)	612 (23.5%)	692 (26.6%)	599 (23.0%)
Children aged 5.5 to 6.5 years old participating in the cross-sectional surveys						
2013	546	5.8 ± 0.2	183 (33.5%)	147 (26.9%)	114 (20.9%)	102 (18.7%)
2014	897	6.0 ± 0.3	239 (26.6%)	236 (26.3%)	219 (24.4%)	203 (22.7%)
2016	512	6.2 ± 0.1	133 (26.0%)	111 (21.7%)	150 (29.2%)	118 (23.1%)

Some descriptive characteristics of families at the start of the study are shown in Table 2. The level of education was higher in the urban mothers than in the rural mothers. Compared to the rural families, the urban families had more assets.

**Table 2.** Socioeconomic characteristics of families at the beginning of study in 2013.

Variables	Urban	Rural
Mother’s education		
Secondary school or less (%)	6.8***	57.6
High school education (%)	32.3***	28.6
Higher than high school (%)	60.9***	13.8
Mother’s occupation		
Manual worker (%)	9.3***	73.4
Office staff (%)	56.7***	9.0
Business (%)	25.5**	14.0
Other (%)	8.5	3.6
Economic level		
Number of family assets in 2013 (mean)	8.8***	5.4

The number of assets (bicycle, motorbike, car, telephone, mobile phone, radio, television, video player, sewing machine, computer, refrigerator, air conditioner, hot water) available in the households was used as an indicator of family economy.

The stars refer to the comparison between the urban and rural population.

**p < 0.01; ***p < 0.001.

Table 3 shows the estimated prevalence of OWOB, stunting, and concurrent OWOB and stunting by place and sex. In the cohort of preschool children followed for 3 years, the prevalence of OWOB increased over time from 11.7% in 2013 to 26.0% in 2016 with a higher rate of increase in the urban children. In general, the prevalence of stunting, and of concurrent OWOB and stunting decreased from 8.8% in 2013 to 3.4% in 2016 and 2.7% to 1.4% respectively, particularly in the rural children. OWOB was more common in urban areas, while stunting, and concurrent OWOB and stunting were more prevalent in the rural areas. These patterns were similar for both boys and girls.

**Table 3.** Estimated prevalence of OWOB, stunting, and concurrent OWOB and stunting by setting and sex in preschool children followed for 3 years (2013 to 2016) using WHO reference.

		OWOB only		Stunting only		Concurrent OWOB and stunting	
		n	% (95% CI)	n	% (95% CI)	n	% (95% CI)
Total studied population (n = 2,602)	2013	306	11.7(10.6–13.0)	229	8.8(7.8–9.9)	69	2.7 (2.1–3.3)
	2014	416	16.0 (14.6–17.4)	175	6.7 (5.8–7.8)	31	1.2 (0.8–1.7)
	2016	677	26.0*** (24.4–27.7)	89	3.4*** (2.8–4.2)	36	1.4*** (1.0–1.9)
Boys total (n = 1,391)	2013	197	14.2 (12.4–16.1)	114	8.2 (6.9–9.8)	34	2.4 (1.8–3.4)
	2014	264	19.0 (17.0–21.1)	87	6.3 (5.1–7.7)	19	1.4 (0.9–2.1)
	2016	416	29.9*** (27.6–32.4)	47	3.4*** (2.6–4.5)	20	1.4 (0.9–2.2)
Urban (n = 699)	2013	167	23.9 (20.9–27.2)	15	2.1 (1.3–3.5)	7	1.0 (0.5–2.0)
	2014	223	31.9 (28.6–35.4)	9	1.3 (0.7–2.4)	4	0.6 (0.2–1.4)
	2016	311	44.5*** (40.8–48.2)	13	1.9 (1.1–3.2)	13	1.9 (1.1–3.2)
Rural (n = 692)	2013	30	4.3 (3.1–6.1)	99	14.3 (11.9–17.1)	27	3.9 (2.7–5.6)
	2014	41	5.9 (4.4–7.9)	78	11.3 (9.1–13.8)	15	2.2 (1.3–3.5)
	2016	105	15.2*** (12.7–18.1)	34	4.9*** (3.5–6.8)	7	1.0*** (0.5–2.1)
Girls total (n = 1,211)	2013	109	9.0 (7.5–10.7)	115	9.5 (7.9–11.2)	35	2.9 (2.1–3.9)
	2014	152	12.6 (10.8–14.5)	88	7.3 (5.9–8.9)	12	1.0 (0.6–1.7)
	2016	261	21.6*** (19.3–23.9)	42	3.5*** (2.6–4.7)	16	1.3** (0.8–2.1)
Urban (n = 612)	2013	91	14.9 (12.3–17.9)	15	2.5 (1.5–4.0)	10	1.6 (0.9–2.9)
	2014	133	21.7 (18.6–25.2)	5	0.8 (0.3–1.9)	5	0.8 (0.3–1.8)
	2016	210	34.3*** (30.7–38.2)	11	1.8 (1.0–3.2)	9	1.5 (0.8–2.8)
Rural (n = 599)	2013	18	3.0 (1.9–4.7)	100	16.7 (13.9–19.9)	25	4.2 (2.8–6.1)
	2014	19	3.2 (2.0–4.9)	83	13.9 (11.3–16.9)	7	1.2 (0.6–2.4)
	2016	51	8.5*** (6.5–11.0)	31	5.2*** (3.7–7.3)	7	1.2** (0.6–2.4)

p-values refer to the comparison with estimates for 2013.

*p < 0.05; **p < 0.01; ***p < 0.001.

Table 4 indicates the prevalence of OWOB, stunting, and concurrent OWOB and stunting in three samples of children aged 5.5 to 6.5 years in the 2013, 2014 and 2016 surveys. The prevalence of OWOB increased significantly among the 3 cohorts of urban girls (from 23.1% in 2013 to 42.3% in 2016) and rural boys (from 11.4% in 2013 to 18.0% in 2016) while it decreased slightly among urban boys and rural girls.

**Table 4.** Estimated prevalence of OWOB, stunting, and concurrent OWOB and stunting by setting and sex in children aged 5.5–6.5 years old in three repeated cross-sectional studies using WHO reference.

		OWOB		Stunting		Concurrent OWOB and stunting	
		n	% (95% CI)	n	% (95% CI)	n	% (95% CI)
Total studied population	2013 (n = 546)	140	25.6 (22.1–29.5)	50	9.2 (7.0–11.9)	17	3.1 (2.0–4.9)
	2014 (n = 897)	201	22.4 (19.8–25.3)	62	6.9 (5.4–8.8)	11	1.2 (0.7–2.2)
	2016 (n = 512)	139	27.1 (23.5–31.2)	25	4.9** (3.3–7.1)	6	1.2* (0.5–2.5)
Boys total	2013 (n = 297)	91	30.6 (25.7–36.1)	28	9.4 (6.6–13.3)	9	3.0 (1.6–5.6)
	2014 (n = 548)	120	26.2 (22.4–30.4)	31	6.8 (4.8–9.4)	6	1.3 (0.6–2.8)
	2016 (n = 283)	81	28.6 (23.7–34.1)	10	3.5** (1.9–6.4)	2	0.7* (0.2–2.5)
Urban	2013 (n = 183)	78	42.6 (35.7–49.9)	8	4.4 (2.2–8.4)	2	1.1 (0.3–3.8)
	2014 (n = 239)	99	41.4 (35.4–47.8)	4	1.7 (0.7–4.2)	2	0.8 (0.2–2.9)
	2016 (n = 133)	54	40.6 (32.6–49.1)	4	3.0 (1.2–7.5)	1	0.8 (0.1–4.1)
Rural	2013 (n = 114)	13	11.4 (6.8–18.5)	20	17.5 (11.7–25.6)	7	6.1 (3.0–12.1)
	2014 (n = 219)	21	9.6 (6.4–14.2)	27	12.3 (8.6–17.3)	4	1.8* (0.7–4.6)
	2016 (n = 150)	27	18.0 (12.7–24.9)	6	4.0*** (1.8–8.4)	1	0.7* (0.1–3.6)
Girls total	2013 (n = 249)	49	19.7 (15.2–25.1)	22	8.8 (5.9–13.0)	8	3.2 (1.6–6.2)
	2014 (n = 439)	81	18.5 (15.1–22.3)	31	7.1 (5.0–9.8)	5	1.1 (0.5–2.6)
	2016 (n = 229)	58	25.3 (20.1–31.3)	15	6.6 (4.0–10.5)	4	1.7 (0.7–4.4)
Urban	2013 (n = 147)	34	23.1 (17.1–30.6)	5	3.4 (1.5–7.7)	2	1.4 (0.3–4.8)
	2014 (n = 236)	73	30.9 (25.4–37.1)	6	2.5 (1.2–5.4)	4	1.7 (0.7–4.2)
	2016 (n = 111)	47	42.3** 33.6–51.6)	4	3.6 (1.4–8.9)	1	0.9 (0.2–4.9)
Rural	2013 (n = 114)	15	14.7 (9.1–22.9)	17	16.7 (10.7–25.1)	6	5.9 (2.7–12.2)
	2014 (n = 219)	8	3.9*** (2.0–7.5)	25	12.3 (8.5–17.5)	1	0.5** (0.08–2.7)
	2016 (n = 150)	11	9.3 (5.3–15.9)	11	9.3 (5.3–15.9)	3	2.5 (0.9–7.2)

p-values refer to the comparison with estimates for 2013.

*p < 0.05; **p < 0.01; ***p < 0.001.

The corresponding secular trend in stunting indicated a decrease between cohorts studied in 2013, 2014 and 2016 although statistical significance only was observed in rural boys. Regarding concurrent OWOB and stunting, a similar decreasing pattern was seen in the total studied population, particularly the rural cohorts of boys showed progressively lower (6.1% to 1.8% to 0.7%) over the 3 surveys.

## Discussion

Findings of both longitudinal and repeated cross-sectional data show that there is an increase in prevalence of overweight and a decrease in prevalence of stunting as well as of concurrent OWOB and stunting. Regarding concurrent undernutrition and overnutrition in Vietnam, the prevalence we found is comparable to other low- and middle-income countries [11,12]. Between 1945 and 1975, Vietnam experienced a long period of food shortage due to war, which was followed by post-war consequences, resulting in a high prevalence of underweight and stunting. The prevalence of underweight among children under 5 years of age was 51.5% in 1985 [13] and still 44.9% 10 years later [13]. Economic development following the renovation policy in 1986 led to a reduction of poverty as well as a general improvement of the health. The percentage of people living in extreme poverty dropped from over 50% in the 1990’s to 3% in 2012 [14]. The prevalence of stunting among children under 5 years of age decreased significantly from 61.3% in 1988 to 23.3% in 2010 [15]. The results of our study provide an update on trends in malnutrition among children, particularly decreases in stunting. The reduction in the prevalence of stunting might be related to the changes of the socio-economic conditions over time. In addition, several programs using media communication, education, training of proper nutrition for communities, programs for school nutrition et cetera, have been successfully implemented to reduce undernutrition [16].

Economic progress has, however, also brought negative effects. Economic growth also introduced unhealthy lifestyles, such as energy-dense diets and increased sedentary behaviour. Traditional Vietnamese diets, where rice is the staple food and the main source of calories, have been replaced by other food types [7]. A survey on preschool children in Ho Chi Minh City in 2005 indicated that 98.1% and 12.7% of the children exceeded the Vietnamese recommendation for energy intake from protein and fat respectively [17]. The higher prevalence of OWOB in urban than in rural children in our study and other studies conducted in Vietnam [7,18–20] reflects these dramatic economic and social changes.

The strength of our research is the combined longitudinal and cross-sectional design allowing us to monitor the prevalence of different forms of malnutrition, both longitudinally during age-related growth and cross-sectionally by comparing different birth cohorts at similar ages. Cross-sectional results of a single age group allow for age-specific comparisons of differences over time. The prevalence of stunting decreased significantly among both boys and girls aged 5.5 to 6.5 years old in the cohort comparisons (from 9.2% in 2013 to 4.9% in 2016) as well as in the longitudinal prevalence changes in individual children followed for three years (8.8% in 2013 to 3.4% in 2016). This parallel development could be evidence of an impact of changing environmental factors on stunting in young children. This may be compared with the slightly increased cross-sectional prevalence of OWOB in children aged 5.5 to 6.5 years old (25.6% in 2013 vs. 27.1% in 2016) as opposed to the remarkably increased prevalence among younger children followed for 3 years (from 11.7% to 26.0%). This comparison is consistent with the hypothesis that OWOB is associated directly with factors at individual child level but elaborated by contextual factors at the community level. A limitation of this study is that the sample sizes of the repeated cross-sectional surveys are generally small and the estimation could only be made for one specific age group. However some differences are statistically significant and important, such as a higher OWOB in urban girls and lower stunting and concurrent OWOB and stunting in rural boys over 3 years. The results may form a basis for future research questions.

Concurrent overweight and stunting was found in this cohort. Theoretically, a decreased length/height growth may contribute to a significant increase of the body mass index (BMI), as it is calculated as weight (kg) divided by squared height (m). Stunting occurs when height growth is hampered because of different causal factors, one of which is inadequate energy intake. If the amount of energy increases, perhaps even to an excess, a child will immediately gain weight and make a catch-up while he or she continues the height growth on a low height percentile and the height catch-up occurs at a slower speed at a later age. As a result, the child will be stunted and overweight simultaneously until the height catch-up has reached a higher level. The higher prevalence of concurrent overweight and stunting in rural children as compared to urban found in this study, is consistent with a study in Ghana [21]. Several studies have found that breastfeeding, socioeconomic status and the habitation of certain geographic areas [21,22] are factors associated with the coexistence of overweight and stunting development.

## Conclusions

Vietnam is experiencing a dual burden of undernutrition and overnutrition with a recent pattern of decreasing stunting and increasing overweight. Children with stunting could be found in certain Vietnamese population groups though the prevalence generally seems to have decreased. However, even so, the presence of overweight and stunting in contemporary Vietnamese at individual and community level highlights the complexity in designing community-based prevention and intervention to reduce underweight and stunting, while, at the same time managing the problem of overweight.

## Paper context

Undernutrition and overnutrition constitute a public health concern in many countries. Children can be overweight and stunted simultaneously. This paper presents results from longitudinal and repeated cross-sectional studies which show that there is an increase in prevalence of overweight, decrease in prevalence of stunting and concurrent overweight and stunting in Vietnam. It highlights the complexity in designing community-based prevention and intervention to reduce underweight and stunting, while managing the problem of overweight.
